# Randomized clinical trial of LigaSure versus conventional suture ligation in thyroid surgery

**DOI:** 10.1186/1758-3284-4-2

**Published:** 2012-01-18

**Authors:** Anandi HW Schiphorst, Bas A Twigt, Sjoerd G Elias, Thijs van Dalen

**Affiliations:** 1 Department of Surgery, Diakonessenhuis, Utrecht, the Netherlands; 2 Julius Center for Health Sciences and Primary Care, University Medical Center Utrecht, the Netherlands

**Keywords:** Operative Surgical Procedures, Thyroid surgery, Blood, Complications, LigaSure, Suture ligation, Operation time

## Abstract

**Background:**

In thyroid surgery vessel division and haemostasis make up an important and time consuming part of the operation. While the presence of the recurrent laryngeal nerve limits the liberal use of diathermia, the many arterial and venous branches to and from the thyroid gland necessitates the use of numerous conventional suture ligatures.This study evaluates the effect of using a vessel sealing system on operation time during thyroid surgery.

**Methods:**

A randomized clinical trial was performed between September 2005 and October 2008 in a teaching hospital. Forty patients undergoing total hemithyroidectomy participated in the trial. Twenty were randomized to the intraoperative use of the LigaSure Precise™ vessel sealing system, and twenty to the use of conventional suture ligatures.

**Results:**

The total median operation time was 10 minutes shorter in the LigaSure group (56 versus 66 minutes, *P *= 0.001). No significant differences in complications were noticed.

**Conclusion:**

Using an electrothermal vessel sealing system during thyroid surgery is time saving.

**Trial registration:**

This trial was registered in the international standard randomized controlled trials number register (ISRCTNR) under number ISRCTNR82389535.

## Background

In thyroid surgery vessel division and haemostasis make up an important and time consuming part of the operation. While the presence of the recurrent laryngeal nerve limits the liberal use of diathermia, the calibre of many arterial and venous branches to and from the thyroid gland necessitates the use of numerous conventional suture ligatures.

Electrothermal bipolar vessel sealing (LigaSure) has proven to be safe and effective for "sealing" medium-sized vessels. A clinically relevant decrease of operative time has been shown in abdominal surgery as well as for haemorrhoidectomy [1-3].

We conducted a randomized trial to assess the effect of LigaSure on operation time in thyroid surgery.

## Methods

**Figure 1:**
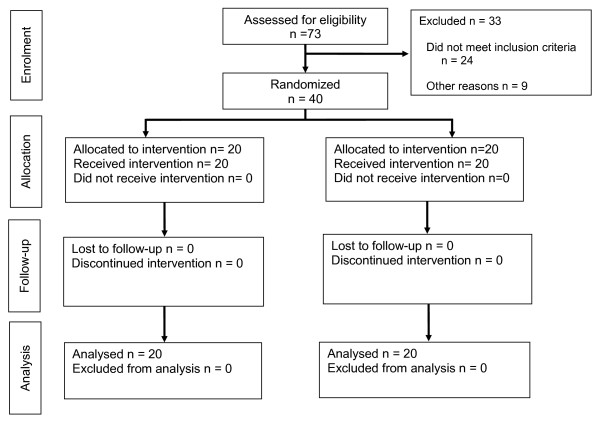
**CONSORT diagram template**.

Following approval of the institutional ethics committee and after having gained experience with the vessel sealing device, the study was initiated (ISRCTN82389535). Between September 2005 and October 2008 forty patients scheduled for a total hemithyroidectomy consented to participate in the study. Twenty-four patients did not meet the inclusion criteria and 9 patients were excluded for other reasons (Figure 1).

Patients were unaware of allocation to either intraoperative use of the LigaSure Precise™ vessel sealing system (Valleylab, Tyco Healthcare, Boulder, Colorado, USA) or conventional suture ligatures. Randomization was done by shuffled sealed envelopes, opened by the surgeon at the start of anaesthesia. The use of diathermia for smaller vessels was allowed in both treatment arms. No surgical clips or other haemostatic aids were used in either group. All operations were performed by a surgical resident and the senior author (ThvD).

Three phases in the operative procedure were defined:

I, Kochers skin incision, separation and undermining of the strap muscles, sealing of middle thyroid veins

II, ligation of the upper thyroid pole vessels and the ima veins, dissection of the lateral part of the gland to the isthmus and cleavage of the gland in the midline

III, haemostasis and closure.

The total operation time measured from the time of incision to the moment of skin closure was the primary endpoint of the study. The duration of the different phases of the operation, the number of sealed or ligated vessels, and postoperative complications (bleeding necessitating re-operation and recurrent laryngeal nerve palsy) were secondary endpoints.

Chi-square and Mann-Whitney U tests were performed to test differences between the groups (SPSS 11 (SPSS, Chicago, IL)). All tests were 2-sided with a 0.05 cut-off for statistical significance. Sample size was determined with PASS Power analysis software (NCSS, Kaysville, Utah, USA) using simulation; the study had a power of 0.85 to detect a 15 minutes difference in median total operation time given a 2-sided alpha of 0.05.

## Results

The median age of the patients was 47 years (22-82 years), there were more female patients (87, 5%). Age, gender, indication for surgical treatment and the operating surgeon (surgical resident or senior surgeon) were evenly distributed (Table 1).

**Table 1:** Patient characteristics

	LigaSure (n = 20)	Conventional (n = 20)	*P*
Age (yrs); median (range)	45 (23-79)	49 (22-82)	N.S.
Gender (male vs female)	3:17	2:18	N.S.
Indication for operation (n)			
Follicular neoplasm	7	9	
Thyroid cyst/node	8	4	
Suspicion of malignancy	0	2	N.S.
Complementary hemithyroidectomy	1	2	
Multinodular goiter	4	3	
Operated by resident as first surgeon (%)	25%	25%	N.S.

The results of the study are listed in Table 2. The median total operation time was 10 minutes shorter in the LigaSure group (56 versus 66 minutes, *P *= 0.001), amounting to a 15% decrease in total operative time over the 40 patients studied. Most time was saved during the second phase of the operation (29 versus 38 minutes, *P *< 0.001). In the LigaSure group, significantly more vascular structures were sealed than in the conventional group (*P <*0.001).

**Table 2:** Operation time, number of ligated vessels and complications in the LigaSure and conventional ligation Group.

	LigaSure (n = 20)	Conventional (n = 20)	*P*
*Primary endpoint*			
Total operation time (min); median (range)	56 (37-70)	66 (48-99)	0.001
*Secondary endpoints*			
Length of operation phases (min); median (range)			
Operation phase I	16 (9-20)	17 (12-28)	N.S.^a^
Operation phase II	29 (17-40)	38 (21-50)	< 0.001
Operation phase III	12 (7-26)	11 (7-16)	N.S.
Number of ligated vessels; median (range)			
Operation phase I	1 (0-3)	1 (0-4)	N.S.
Operation phase II	14 (8-32)	8 (3-12)	< 0.001
Operation phase III	3	0	N.S.
Total	16 (8-34)	9 (4-16)	< 0.001
Number of patients with complicated postoperative course	2	0	N.S.

No patient needed a reoperation for postoperative haemorrhage. Two patients in the LigaSure group suffered from transient recurrent laryngeal nerve palsy that resolved spontaneously in both patients after six and seven weeks respectively. This difference was not significant.

## Discussion

In this randomized trial a significant time saving effect of the LigaSure vessel sealing system was observed in thyroid surgery. A median 10 minutes were gained during hemithyroidectomy. As most of the time was saved during the actual dissection of the lobe, this would potentially result in a twenty minutes time saving when a total (bilateral) thyroidectomy is done.

**Figure 2:**
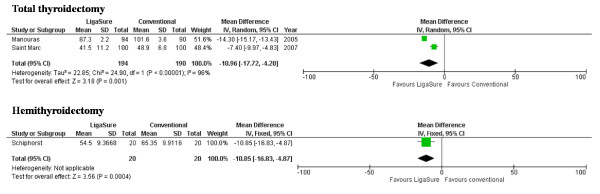
**Forest plot of randomized trials measuring operative time in thyroid surgery (Ligasure versus conventional suture ligations)**.

The main weakness of the present study is the limited number of patients. Two other randomized trials with larger patient numbers have been published during the period of patient inclusion of the present study [4,5]. In both trials a total, i.e. bilateral thyroidectomy, was done, and both studies reported a time saving effect when the LigaSure was used (see Figure 2). Saint Marc et al. concluded however that the time saving was clinically hardly relevant: 41 minutes when using the ligasure versus 49 when using conventional suture ligations [5]. Although this absolute gain of approximately seven and a half minute does not appear to be clinically significant, the total operative time has to be taken into account. In their study, the reported operative time for a total (bilateral) thyroidectomy is extremely short and is not in line with other randomised or prospective studies, reporting a total operation time for a total thyroidectomy between 70-120 minutes [4,6,7]. Furthermore in both other trials haemostatic clips were used in both treatment arms too.

The safety of the Ligasure device is another important issue. We observed two patients with postoperative hoarseness in the Ligasure-arm. In both patients the hoarseness resolved spontaneously within seven weeks. The present trial was not designed or powered to demonstrate a difference or equivalence of safety, but in the aforementioned other trials there was no difference in frequency of recurrent nerve paralysis or postoperative bleeding [4,5]. A recent meta-analysis showed that electrothermal bipolar vessel sealing systems are safe and efficient in other fields of surgery (even laparoscopic surgery) [8].

In conclusion, using LigaSure in thyroid surgery leads to a clinically relevant saving of operative time.

## Competing interests

The authors declare that they have no competing interests.

## Authors' contributions

AS carried out the collection of patient data and drafted the manuscript. BT helped in coordinatimg the trial, acquisition of data and helped to draft the manuscript. SE participated in the design of the study, analysis and interpretation of data and performed the statistical analysis. TvD conceived and designed the study and helped to draft the manuscript. All authors read and approved the final manuscript.

## References

[B1] Lee WJ Chen TC Lai IR Wang W Huang MT Randomized clinical trial of Ligasure versus conventional surgery for extended gastric cancer resection Br J Surg 2003 90 12 1493 6 10.1002/bjs.4362 14648726

[B2] Muzi MG Milito G Nigro C Cadeddu F Andreoli F Amabile D Farinon AM Randomized clinical trial of LigaSure and conventional diathermy haemorrhoidectomy Br J Surg 2007 94 937 942 10.1002/bjs.5904 17636512

[B3] Saiura A Yamamoto J Koga R Sakamoto Y Kokudo N Seki M Yamaguchi T Yamaguchi T Muto T Makuuchi M Usefulness of LigaSure for liver resection: analysis by randomized clinical trial Am J Surg 2006 192 41 45 10.1016/j.amjsurg.2006.01.025 16769273

[B4] Manouras A Lagoudianakis EE Antonakis PT Filippakis GM Markogiannakis H Kekis PB Electrothermal bipolar vessel sealing system is a safe and time-saving alternative to classic suture ligation in total thyroidectomy Head Neck 2005 27 959 962 10.1002/hed.20271 16134183

[B5] Saint Marc O Cogliandolo A Piquard A Famà F Pidoto RR LigaSure vs clamp-and-tie technique to achieve hemostasis in total thyroidectomy for benign multinodular goiter: a prospective randomized study Arch Surg 2007 142 150 156 discussion 157 10.1001/archsurg.142.2.150 17309966

[B6] Kirdak T Korun N Ozguc H Use of ligasure in thyroidectomy procedures: results of a prospective comparative study World J Surg 2005 29 771 774 10.1007/s00268-005-7788-y 15883664

[B7] Petrakis IE Kogerakis NE Lasithiotakis KG Vrachassotakis N Chalkiadakis GE LigaSure versus clamp-and-tie thyroidectomy for benign nodular disease Head Neck 2004 26 903 909 10.1002/hed.20073 15390199

[B8] Macario A Dexter F Sypal J Cosgriff N Heniford BT Operative time and other outcomes of the electrothermal bipolar vessel sealing system (LigaSure) versus other methods for surgical hemostasis: a meta-analysis Surg Innov 2008 15 284 291 10.1177/1553350608324933 18945705

